# Nonlinear progression during the occult transition establishes cancer lethality

**DOI:** 10.1242/dmm.052113

**Published:** 2025-03-19

**Authors:** Joshua D. Ginzel, Henry Chapman, Joelle E. Sills, Edwin J. Allen, Lawrence S. Barak, Robert D. Cardiff, Alexander D. Borowsky, Herbert Kim Lyerly, Bruce W. Rogers, Joshua C. Snyder

**Affiliations:** ^1^ Department of Surgery, Duke University, Durham, NC 27710, USA; ^2^ Department of Cell Biology, Duke University, Durham, NC 27710, USA; ^3^ Department of Pathology and Laboratory Medicine, UC Davis, Davis, CA 95817, USA; ^4^ Department of Pharmacology and Cancer Biology, Duke University, Durham, NC 27710, USA

**Keywords:** Breast cancer, Mathematical modeling, Cancer progression, Systems biology, Quantitative biology, Mouse models of cancer

## Abstract

Cancer screening relies upon a linear model of neoplastic growth and progression. Yet, historical observations suggest that malignant progression is uncoupled from growth, which may explain the paradoxical increase in early-stage breast cancer detection without a dramatic reduction in metastasis. Here, we lineage trace millions of transformed cells and thousands of tumors using a cancer rainbow mouse model of HER2 (also known as ERBB2)-positive breast cancer. Transition rates from field cell to screen-detectable tumor to symptomatic tumor were estimated from a dynamical model of tumor development. Field cells were orders of magnitude less likely to transition to a screen-detectable tumor than the subsequent transition from screen-detectable tumor to symptomatic tumor. Our model supports a critical ‘occult’ transition in tumor development during which a transformed cell becomes a bona fide neoplasm. Lineage tracing and test by transplantation revealed that nonlinear progression during the occult transition gives rise to nascent lethal cancers at screen detection. Simulations illustrated how occult transition rates are a critical determinant of tumor growth and malignancy. Our data provide direct experimental evidence that cancers can deviate from the predictable linear progression model that is foundational to current screening paradigms.

## Teaser

**Summary:** Experimental and mathematical modeling reveals that the transition from a transformed cell to an invasive tumor occurs independently from screen detection, challenging the existing paradigm in breast cancer screening.

## INTRODUCTION

Progression of a screen-detectable early-stage tumor to a symptomatic and metastatic tumor is often regarded as the pivotal moment in the natural history of cancer (Gil Del Alcazar et al., 2017). As such, most cancer screens are focused on identifying and treating small tumors prior to their growth and progression to metastasis. Although this concept has led to remarkable progress in cancer-screening programs, the costs and benefits of screening are being questioned (Welch et al., 2019). For instance, not all screen-detectable ductal carcinomas of the breast progress to invasive ductal carcinoma, and, despite significant progress in early detection, the metastatic burden remains relatively unchanged (Welch et al., 2019, 2016).

Leslie Foulds proposed six rules of tumor progression more than 70 years ago. Foulds' rules conceptualized that progression to malignancy occurs independent of neoplastic growth, that it can occur by multiple developmental trajectories, and that it can occur abruptly or gradually (Foulds, 1969, 1958, 1957). Thus, it should not be surprising how screening for early-stage tumors by size alone may not produce optimal benefits. Yet, the absence of quantitative proof of Foulds' rules in immune intact mammalian models continues to confound our understanding of critical inflection points in the disease process. One major challenge is that most of the disease process occurs over the course of several years in humans and is dependent upon cancer cell-extrinsic adaptations in the tumor microenvironment (Gerstung et al., 2020; Swanton et al., 2024). Tumor driver gene mutations also occur several years prior to detection, and many fully transformed clones can stall or extinguish entirely without ever causing disease (Brown et al., 2017; Hill et al., 2023; Huels et al., 2018; van Neerven et al., 2021). As a result, for every transformed cell that develops into cancer, billions of transformed cells exist in healthy tissues that may never form a tumor in a patient's lifetime (Evans and DeGregori, 2021). Altogether, this makes solving for the tumorigenic potential of a transformed cell and its progression to a metastatic cancer extremely challenging in a complex biological organism.

We previously developed cancer rainbow mouse (Crainbow) models for fluorescently visualizing and barcoding premalignant clones (Boone et al., 2019; Ginzel et al., 2021). Our previous work in the mammary gland validated a HER2 Crainbow (HBOW) model that expresses multiple clinically relevant isoforms of the protooncogene *HER2* (also known as *ERBB2*), including the wild-type (WT) isoform, an exon 16 splice isoform (d16) and an N-terminally truncated isoform (p95) (Ginzel et al., 2021). We qualitatively found that the clinically apparent and symptomatic phase of tumorigenesis in HBOW mice is characterized by isoform-dependent differences in tumor phenotypes. Lineage tracing and histology revealed that these isoform-dependent differences occur even in the smallest detectable hyperplastic lesions, suggesting that, even in mouse models, an early inflection point in the disease process must occur prior to detection. However, quantitative understanding of tumor development and progression requires hundreds to thousands of samples across the entire course of the disease.

Our goal here was to lineage trace and mathematically model tumor growth in order to answer several basic questions that remain unresolved. How many genetically transformed cells in a field are required to form a tumor, how frequently do transformed cells establish screen-detectable tumors, and how frequently do these screen-detectable tumors transition to clinically apparent and symptomatic tumors? Lastly, we sought to determine whether progression occurs independent of these growth phases. We demonstrate that an ‘occult’ transition from a genetically transformed cell to a bona fide tumor is exceptionally rare and is the rate-limiting step in the growth of a tumor. Our data also show that dissemination and metastasis do not necessarily correlate with growth rate and further illustrate how an abrupt progression to malignancy during the occult transition establishes nascent lethal cancers prior to detection.

## RESULTS

**Fig. 1.**
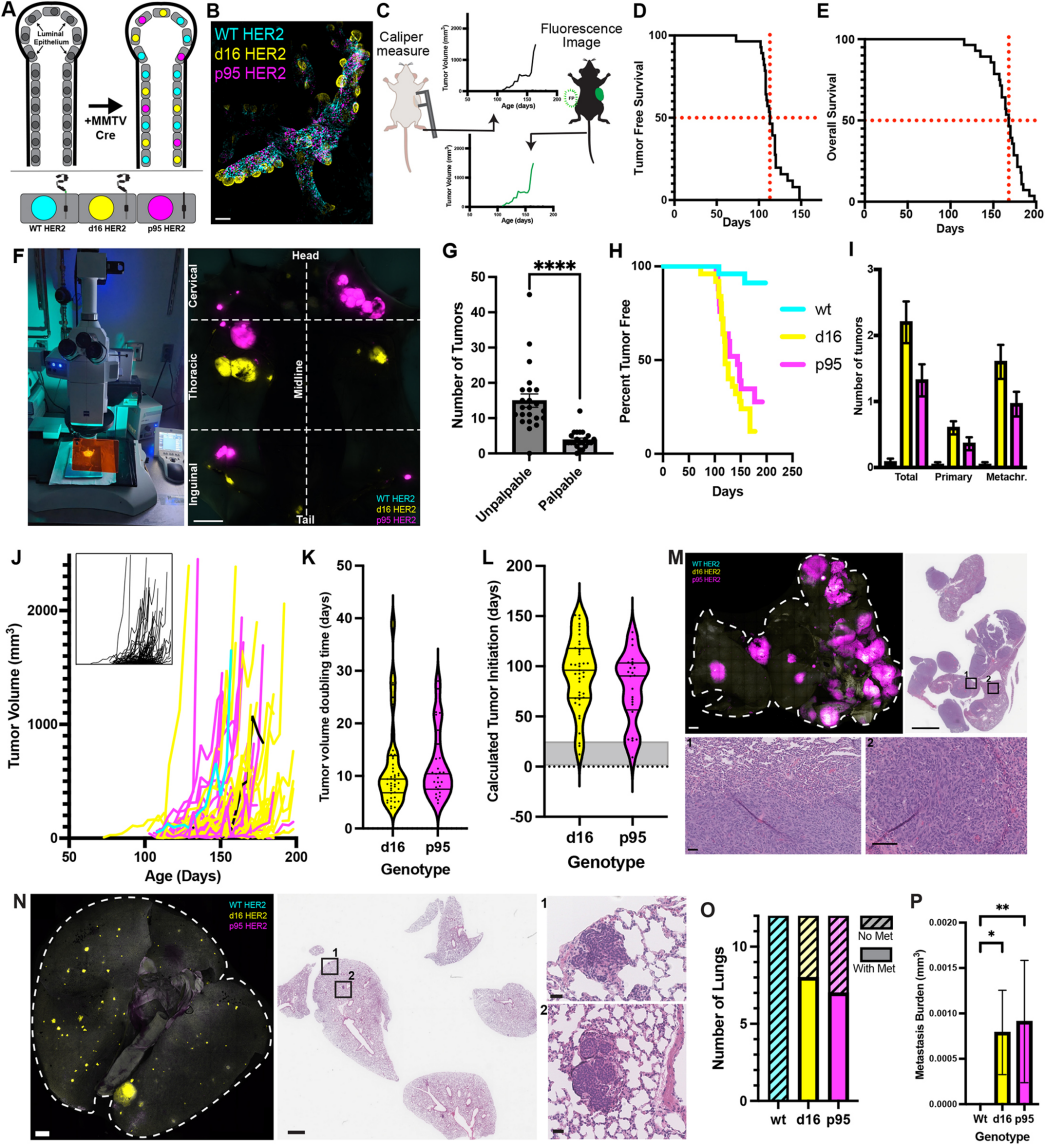
**Quantifying the visible phase of tumorigenesis.** (A) Schematic of HER2 Crainbow (HBOW) activation in the luminal epithelium of the mammary gland in the presence of MMTV Cre. Upon Cre recombination, luminal epithelial cells stochastically express either mTFP1:WT HER2 (cyan), eYFP:d16 HER2 (yellow) or mKO:p95 HER2 (magenta). (B) Whole-mammary gland imaging of fluorescent Crainbow cells at 2 weeks. (C) Tumors palpable through the skin were measured twice weekly with calipers until the tumor burden endpoint. Mice were imaged at necropsy to retrieve fluorescent protein (FP) expression and registered to the palpation data. (D) Tumor-free survival of 28 mice palpated biweekly. Red lines represent 50% on the *y*-axis and 113 days on the *x*-axis. (E) Survival until tumor burden endpoint of 28 mice. Red lines represent 50% on the *y*-axis and 168 days on the *x*-axis. (F) Whole-mouse imaging reveals tumor genotype by fluorescence at necropsy. (G) Number of tumors per mouse measured by whole-mouse imaging of 28 mice that were palpable or too small to palpate (unpalpable). Statistical significance was determined by Mann–Whitney test. ****P<0.0001. (H) Tumor-free survival calculated by the time to the first palpable tumor per mouse of each color as determined by whole-mouse imaging for 28 mice. (I) Number of tumors expressing each HER2 isoform per mouse at tumor burden endpoint (*n*=28 mice). From left to right: total tumors observed, primary tumors observed and all metachronous tumors observed. (J) Observed tumor volume growth over time colored by genotype for 91 tumors. The inset shows growth before Crainbow registration. (K) Tumor volume-doubling time (TVDT) of each observed tumor by genotype (*n*=27 p95 and 45 d16 tumors). Tumors with <3 measurements were not included. (L) Calculated time of tumor initiation from TVDT and first palpation observation (*n*=24 p95 and 41 d16 tumors). Gray shading indicates actual recombination and oncogene expression between 0 and 14 days old. Upper and lower lines in the violin plots in K,L represent the interquartile range and the middle line represents the median. (M,N) Representative three-dimensional whole-lung imaging showing p95 HER2 (M) or d16 HER2 (N) tumor metastasis paired with Hematoxylin and Eosin staining. The lung volume is outlined with a white dotted line. High-magnification insets (1 and 2) are shown for each. (O) The number of lungs with at least one metastatic lesion expressing mTFP1:WT HER2 (cyan), eYFP:d16 HER2 (yellow) or mKO:p95 HER2 (magenta) from mice (*n*=12) at tumor endpoint. (P) Average area of metastatic burden per lung (*n*=12) for each genotype. Statistical significance was determined by Kruskal–Wallis test. *P<0.05; **P<0.01. Error bars are represented as mean±s.e.m. Scale bars: 100 μm for whole-mammary gland image (B); 10 mm for whole mouse (F); 1 mm for whole lung (M,N); 100 μm for insets (M,N).

Tumorigenesis begins at cell transformation when a tumor driver gene is first expressed. In our model, MMTV Cre expression in the luminal epithelium causes recombination and expression of one of three HER2 isoforms paired with a fluorescent protein barcode [mTFP1:WT HER2 (cyan), eYFP:d16 HER2 (yellow) or mKO:p95 HER2 (magenta); Ginzel et al., 2021] throughout the mammary gland (Fig. 1A). Cre recombination and initiation of HER2 expression occurs no later than 2 weeks of age in the mammary rudiment (Fig. 1B). Our goal was to develop a model of tumor development that includes growth of transformed cells, the transition to screen-detectable neoplasms, and then to clinically detectable symptomatic tumors. Therefore, we first conducted a longitudinal study to determine the duration of the preclinical phase and symptomatic phase of tumorigenesis in HBOW mice. We used palpation as a proxy for clinical detection and surveyed HBOW mice for the first sign of a tumor. Then tumors were measured at least two times weekly until the total tumor burden reached 2000 mm^3^ or a humane endpoint. At endpoint, mice were imaged to retrieve the fluorescent protein barcode for each palpable tumor (Fig. 1C). Tumor-free survival and overall survival were both calculated in 28 mice. HBOW mice had a 50% tumor-free survival at 113 days (∼16 weeks of age) and all mice developed tumors by 148 days (∼21 weeks of age) (Fig. 1D). 50% of HBOW mice reached tumor burden by 168 days (∼24 weeks of age) and all mice reached tumor burden by 198 days (∼28 weeks of age) (Fig. 1E). Thus, the preclinical phase of tumorigenesis as measured from the time of oncogene expression to the first symptomatic tumor was approximately 14 weeks, whereas the clinical or symptomatic phase of tumorigenesis was approximately 55 days (7.8 weeks), demonstrating that over two-thirds of the tumor growth phase was missed by palpation only.

All mice were imaged using an epifluorescence dissecting microscope and palpable tumors were co-registered with tumor genotype using the Crainbow fluorescent protein barcode (Fig. 1F). This allowed us to also determine whether differences in the length of the preclinical phase or symptomatic phase could be detected for each HER2 isoform. Whole-mouse imaging allowed for the detection of a significant reservoir of preclinical lesions, undetectable by palpation, that were approximately 3-fold more abundant than palpable lesions and as small as 0.5 mm in diameter (Fig. 1G). Of the 90 palpable tumors, there were two WT HER2-expressing tumors (visualized by mTFP1 fluorescence in cyan), 55 d16 HER2-expressing tumors (eYFP fluorescence in yellow) and 33 p95 HER2-expressing tumors (mKO fluorescence in magenta). Using this registration, tumor-free survival was plotted for each HER2 isoform. 20 of the surveyed mice had at least one palpable d16 tumor with a median tumor-free survival of 120 days (∼17 weeks) compared to the 17 mice with at least one palpable p95 tumor and an increased median tumor-free survival of 144 days (∼20 weeks) (Fig. 1H). On average, there were 2.2 d16 tumors, 1.32 p95 tumors and 0.08 WT HER2 tumors per mouse (Fig. 1I). HBOW mice develop multifocal tumors, so we also reported on the lineage of first palpable tumors and compared this to the lineage of metachronous palpable tumors (Ginzel et al., 2021). The first palpable tumors (primary) accounted for 27% of the total tumor burden, whereas the remaining 73% were metachronous tumors that developed independently of the first tumor. Similar frequencies for tumor penetrance for each genotype were observed for primary palpated tumors and metachronous tumors (Fig. 1I). Altogether, these data demonstrate that the length of the preclinical phase is similar for the d16 and p95 isoforms and also confirm that WT HER2 has limited tumorigenic potential.

Next, growth rates were calculated for d16 and p95 tumors. WT HER2 tumors were not included due to their limited number. For each tumor palpated over time, measurements were plotted and registered to the genotype (Fig. 1J). Tumor volume-doubling time (TVDT) is a commonly used tool to assess tumor growth rate and has been correlated to tumor phenotype (Förnvik et al., 2016; Kay et al., 2019). The TVDT was on average 12 days for both p95 and d16 HER2 tumors (Fig. 1K). Assuming that the TVDT is constant, the age of tumor initiation can be calculated by using the first palpable measurement and the calculated TVDT. The age of tumor initiation was inferred to be 93 days (13 weeks) for d16 tumors and 78 days (11 weeks) for p95 tumors (Fig. 1L). However, in our system, oncogene initiation and cell transformation occurred no later than 14 days of age. Thus, palpation and TVDT alone are a poor predictor of tumor initiation and inadequate methods to measure tumor development. Similar to in humans, the majority of the disease process occurred prior to clinical detection and as such could not be estimated from growth during the clinical phase.

The metastatic potential was also measured by three-dimensional whole-lung imaging at the tumor burden endpoint (*n*=12). Representative images show high p95 and d16 metastatic burden in the lungs of two different mice (Fig. 1M,N). Large p95^+^ lung metastases were visualized in an HBOW mouse lung (Fig. 1M). Hematoxylin and Eosin (H&E) staining revealed multiple large nodules of neoplastic cells with fusiform nuclei and scant polar cytoplasm, which appeared to invade and compress adjacent pulmonary tissue (Fig. 1M, insets 1 and 2). A representative image of a lung with d16 HER2 metastasis revealed multiple metastatic proliferative nodules disrupting normal lung architecture, with densely packed hyperchromatic nuclei within small, peripheral vessels (Fig. 1N, insets 1 and 2). Of the 12 lungs analyzed, none had WT HER2 metastasis, eight had d16 metastases and seven had p95 metastases (Fig. 1O). Quantification of the average area of metastatic lesions from whole-lung images revealed that the metastatic burdens for d16 and p95 were similar (Fig. 1P) and demonstrated that each isoform has the potential to drive tumors that progress to metastasis.

**Fig. 2.**
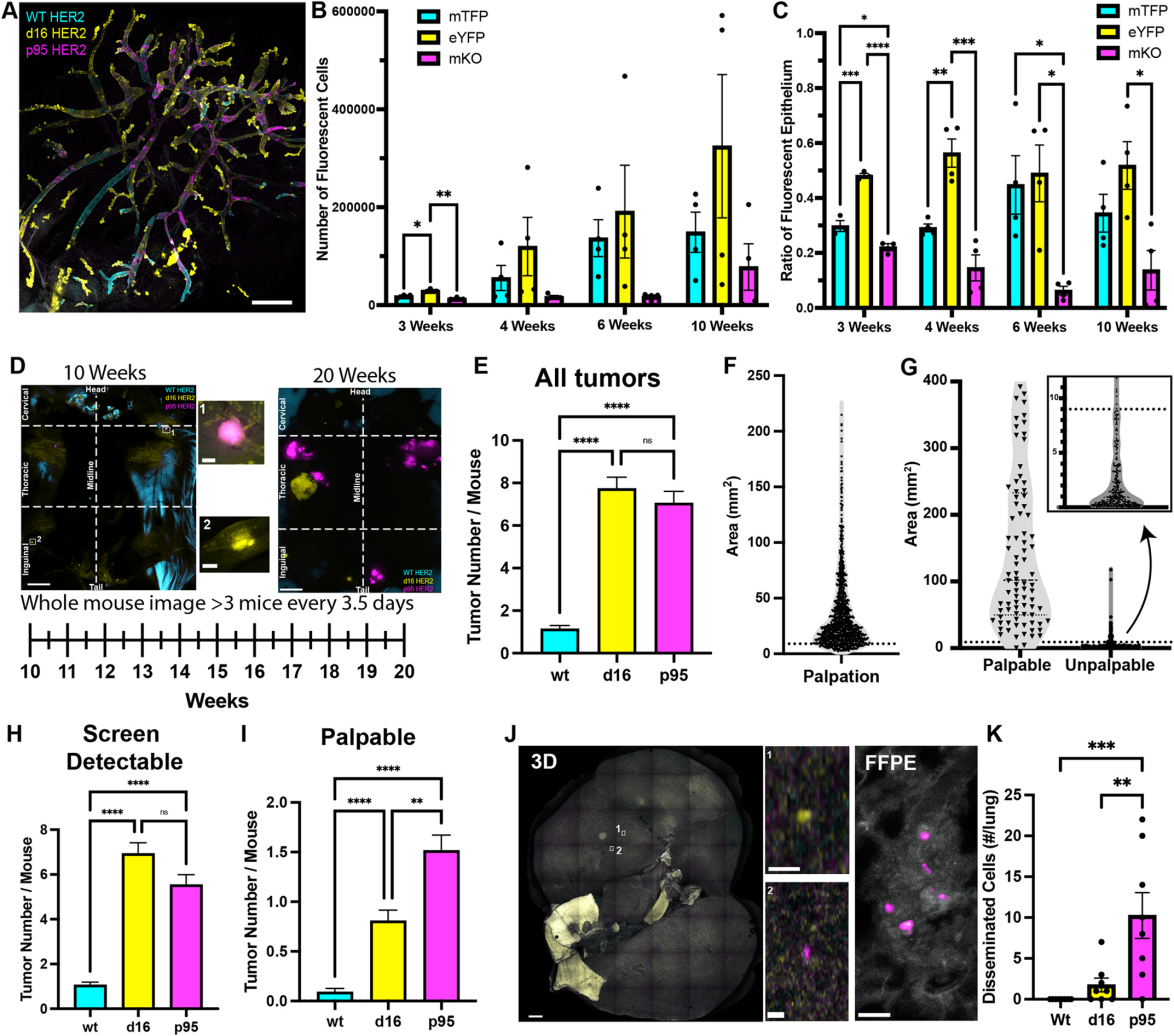
**Quantifying the invisible phase of tumorigenesis**. (A) Whole-mammary gland imaging of fluorescent Crainbow cells expressing mTFP1:WT (cyan), eYFP:d16 (yellow) and mKO:p95 (magenta) HER2 at 10 weeks of age. (B) Quantification of the number of fluorescent cells from all ten mammary glands of HBOW mice after dissociation to single cells at 3, 4, 6 and 10 weeks of age (*n*=3 mice at 3 weeks and 4 mice at 4, 6 and 10 weeks). (C) Ratio of cell number for each genotype to the total number of oncogenic epithelial cells from B. (D) Whole-mouse imaging of HBOW mice at 10 and 20 weeks of age shows representative tumor number and size. To survey occult tumor growth, HBOW mice were sacrificed every 3.5 days from 10 to 20 weeks of age to survey tumor size and genotype. Insets 1 and 2 are high-magnification views from the 10-week-old whole-mouse image. (E) Quantification by genotype of all tumors based on Crainbow fluorescence from whole-mouse imaging (*n*=120 mice). (F) Calculated area of each palpation measurement taken in Fig. 1D,E (*n*=963 tumors). The dotted line marks the 15th percentile at 9 mm^2^. (G) Tumor area from whole-mouse imaging of palpated mice in Fig. 1F,G. The dotted line marks the putative cutoff between palpable and unpalpable tumors at 9 mm^2^. The inset shows the number of unpalpable lesions that fell below the 9 mm^2^ cutoff. (*n*=963 palpable and 344 unpalpable tumors). Dotted lines within the violin plots show the interquartile range and the dashed line shows the median. (H,I) Quantification by genotype of screen-detectable tumors less than 9 mm^2^ (H) and palpable tumors greater than 9 mm^2^ (I) in size based on Crainbow fluorescence from whole-mouse imaging (*n*=120 mice). (J) Representative image from whole-lung imaging of HBOW mice at 10-12 weeks of age. Inset: high-magnification imaging of formalin-fixed paraffin-embedded (FFPE) samples shows single disseminated cells in the lung expressing p95 (magenta) or d16 (yellow). (K) Quantification of the number of disseminated cells from whole-lung imaging at 10-12 weeks of age (*n*=8). Statistical significance was determined by one-way ANOVA with Bonferroni's multiple comparison test (B,C,K) or Kruskal–Wallis Test (E,H,I). ns, not significant; *P<0.05; **P<0.01; ***P<0.001; ****P<0.0001. Error bars are represented as mean±s.e.m. Scale bars: 1 mm (A,J); 10 mm (D); 0.4 mm (D, insets); 20 μm (J, insets, FFPE).

The clinical or symptomatic phase appeared similar for d16 and p95 tumors; the tumors for each genotype grew at similar rates and were capable of progression to a metastatic cancer. However, major differences in the preclinical phase have been overlooked and preclude a quantitative assessment of the actual tumorigenic potential of a single HER2-transformed cell. Simply stated, do d16- and p95-transformed cells grow and progress similarly? Whole-mount imaging of a HBOW mammary gland showed qualitative differences in cell number and phenotype between HER2 isoform-expressing cells at 10 weeks of age (Fig. 2A). We reasoned that we would need to know the number of initial cells transformed, their growth as fields over time, and the rate of production for early screen-detectable tumors (unpalpable) and symptomatic tumors (palpable).

First, we began by collecting data on the number of transformed cells at initiation, their growth and the overall carrying capacity of transformed cells in the mammary gland prior to screen detection. Quantitative imaging and analysis were performed in HBOW mice sacrificed at 3, 4, 6 and 10 weeks of age. Single-cell suspensions from every gland (ten glands/mouse) were prepared and a representative sample of cells counted by confocal microscopy to estimate the total number of cells expressing the different HER2 isoforms. The total carrying capacity, i.e. the number of fluorescent epithelial cells present within the duct, increased by almost 9-fold from 3 weeks to adulthood as expected due to the pubertal development of the mammary duct (Fig. 2B). The numbers of WT and d16 HER2-expressing cells appeared to increase more rapidly from 3 to 6 weeks, whereas p95-expressing cell numbers remained stable until 10 weeks (Fig. 2B). However, there was significant variability in the total number of cells isolated within samples, making comparisons with cell number challenging. When the number of cells expressing each isoform was normalized to the total number of fluorescent cells isolated from mammary glands from each individual, significant trends could be observed (Fig. 2C). Throughout gland development, d16 HER2-expressing cells made up roughly half of the recombined mammary epithelium, whereas WT HER2-expressing cells increased from 30% to 44% during pubertal development (Fig. 2C). In contrast, p95 HER2-expressing cells decreased from 22% to 7% of the recombined epithelium during puberty and variably increased to 14% at 10 weeks of age (Fig. 2C).

Next, we quantified the number of screen-detectable tumors by examining the total number of small, unpalpable lesions over time that were only detectable by whole-mouse imaging. HBOW mice were necropsied and imaged at time points every 3-4 days from 10 to 20 weeks of age to quantify unpalpable, image-detectable lesions immediately after the carrying capacity of the field was reached (10 weeks) and up to the median time of clinical detection (20 weeks) (Fig. 2D). Representative images at 10 weeks show a few small lesions within the HBOW-labeled mammary ducts (Fig. 2D, insets 1 and 2). These lesions continued to increase in number and size through to 20 weeks (Fig. 2D). The image-detectable tumor burden for the d16 HER2 and p95 HER2 isoforms was nearly identical, and each was 7-fold higher than that for WT HER2 (Fig. 2E). In order to differentiate between an early palpable lesion and an unpalpable one from whole-mouse imaging alone, volume measurements from the previous palpation experiments were converted to area and used to find the lower 15th percentile of palpable tumors (9 mm^2^) (Fig. 2F). Applying this cutoff to the whole-mouse imaging data from Fig. 1F,G revealed that 98% of palpable tumors and 88% of unpalpable tumors were correctly classified (Fig. 2G). After stratification of whole-mouse imaging data using the 9 mm^2^ cutoff, mice had on average one WT HER2 tumor, seven d16 HER2 tumors and 5.5 p95 HER2 tumors that were smaller than 9 mm^2^ (screen-detectable, unpalpable) and 0.09 WT HER2 tumors, 0.8 d16 tumors and 1.5 p95 tumors that were larger than 9 mm^2^ (large, palpable) (Fig. 2H,I). Despite having a significantly lower carrying capacity in the field, the p95 isoform exhibited an equivalent number of screen-detectable tumors.

The invasive potential of a breast cancer clone can sometimes be established before screen detection (Casasent et al., 2018; Lips et al., 2022; Wang et al., 2023), resulting in early dissemination and metastasis prior to detection (Harper et al., 2016; Hu et al., 2020; Klein, 2020, 2009). Whole-lung imaging of HBOW cells was performed to quantify the number of HBOW cells that disseminated to the lung (Fig. 2J). Single disseminated cells could be found before palpable tumors could be observed (between 10 and 12 weeks of age) and p95 HER2-expressing cells were 5-fold more numerous in the lung than d16 HER2-expressing cells (Fig. 2J,K). Thus, progression to malignancy is not directly correlated with tumor size and, for p95, it may occur abruptly, prior to screen detection.

**Fig. 3.**
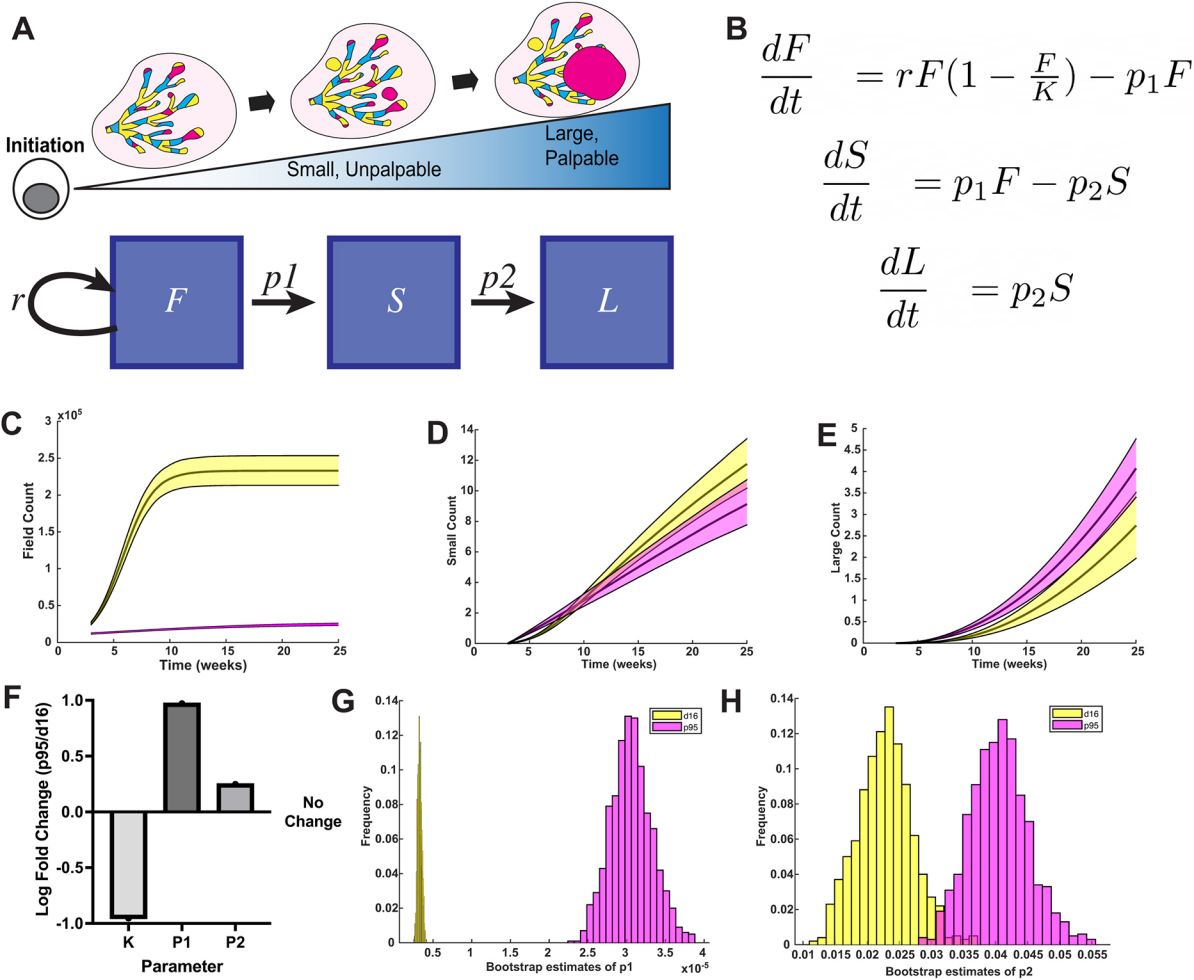
**Three-compartment model of tumorigenesis.** (A) Schematic of discrete states of tumorigenesis in HBOW mice. Fields of recombined cells (*F*) grow at a rate (*r*) before progressing to small, unpalpable lesions (*S*) at rate *p*_1_. Small lesions then progress to large, palpable tumors (*L*) at rate *p*_2_. (B) Ordinary differential equations defining progression rates from one compartment to the next. *K*, field size. (C) Estimated logistic growth of fields of cells expressing d16 (yellow) or p95 (magenta) in HBOW mice over time. (D,E) Logarithmic fit of the number of screen-detectable (*S*) lesions (D) and palpable (*L*) lesions (E) per mouse across time. (F) Fold difference between p95 HER2 and d16 HER2 tumors in field size (*K*), *p*_1_ rate and *p*_2_ rate. (G,H) Bootstrap estimates of *p*_1_ (G) and *p*_2_ (H). In C-E, error bars represent 90% confidence intervals.

We reasoned that our data could be used to quantify the rates of major transitions during tumor development so that we could quantitatively compare growth models and progression potentials for each genotype. Tumors grow along a continuum from the earliest initiation event (*F*), to small, image-detectable lesions (*S*), and finally to large, palpable tumors (*L*) (Fig. 3A). Although we cannot directly trace the growth of a single cell or tumor from start to finish, an ordinary differential equation (ODE) model fitted to static data from many distinct points in time can infer tumor transitions from state to state. Based on the assumptions that all small tumors arise from a transformed cell in the field and that all large tumors were once small tumors, a series of ODEs was defined to capture the surveyed data from Fig. 2 and estimate transition rates from one state to the next (Fig. 3A,B). Owing to the limited number of WT HER2 tumors, we once again excluded them from this analysis. Based on measurements from Fig. 2B, a logistic growth model of the fields of HER2-expressing cells (*F*) was used to describe the exponential growth rate of HER2^+^ cells (*r*) that occurs during puberty from 4 to 6 weeks of age and then approaches a carrying capacity within the mammary duct (*K*) (Fig. 3B,C). d16-transformed field cells expand at an ∼5-fold higher rate than p95-transformed field cells, leading to a 9-fold higher carrying capacity (Fig. 3C). The numbers of small and large tumors over time were plotted by fitting the ODEs to the whole-mouse tumor measurements with 90% confidence intervals (Fig. 3D,E). d16 screen-detectable tumors were more prevalent over time compared to p95 screen-detectable tumors (Fig. 3D). In contrast, the number of large p95 HER2 tumors remained higher than that for d16 HER2 tumors throughout the observed time frame (Fig. 3E).

Our model was then used to determine the state transition that described the high p95 tumor burden despite the lack of a large field. Bootstrapping was used to make statistical inferences and estimate progression rates. The progression rate (*p*_1_) from a transformed cell (*F*) to a screen-detectable tumor (*S*) was a log order higher for p95 HER2 compared to that for d16 HER2 (Fig. 3F,G, Table 1). The progression rate (*p*_2_) from small (*S*) to large tumors (*L*) was also 2-fold higher for p95 HER2 (Fig. 3F,H, Table 1). Comparison of the progression rates between compartments demonstrates how each successive state transition becomes more likely. The model demonstrates that progression from a transformed field cell to a screen-detectable tumor is the rate-limiting step in tumorigenesis and that this transition is much more likely for p95- compared to d16-transformed cells.

**Table 1.** Three-compartment model parameters with 95% confidence intervals

Parameter	d16	95% c.i.	p95	95% c.i.
*F*_0_	26,000	N/A	12,000	N/A
*K*	233,000	N/A	26,000	N/A
*r*	0.7313	N/A	0.1346	N/A
*p*_1_*	3.259×10^−6^	2.786×10^−6^, 3.846×10^−6^	3.048×10^−5^	2.544×10^−5^, 3.599×10^−5^
*p*_2_*	0.0229	0.0148, 0.0315	0.0405	0.0321, 0.0502

*F*_0_, field size at initiation (number of cells); *K*, field carrying capacity (number of cells); *r*, field replacement rate; *p*_1_, transition rate from field to small; *p*_2_, transition rate from small to large.

*Denotes that the 95% confidence intervals generated by bootstrapping do not overlap.

**Fig. 4.**
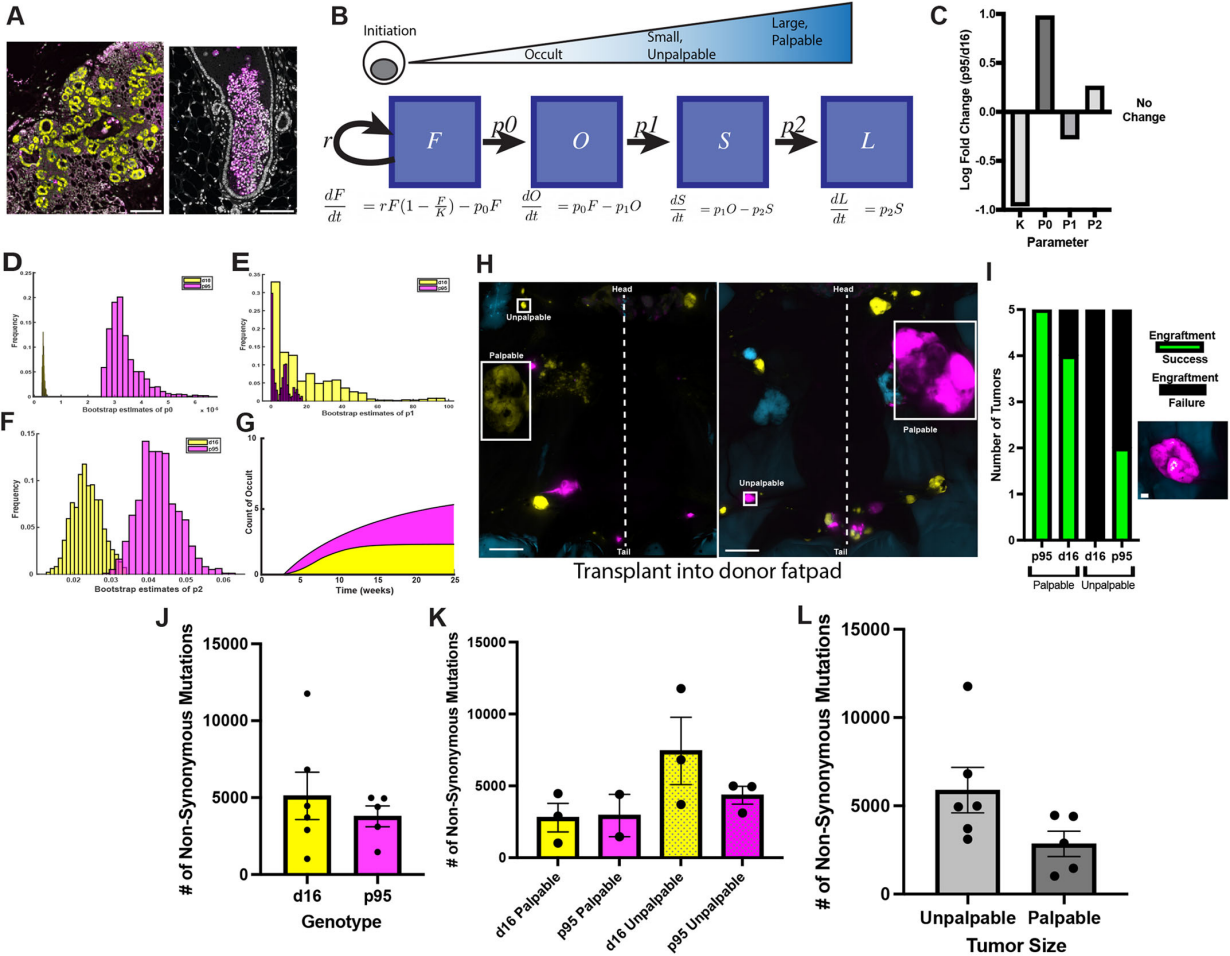
**Four-compartment model of occult tumorigenesis.** (A) Confocal imaging of potential occult lesions in HBOW mammary glands expressing d16 (yellow) or p95 (magenta). Scale bar: 100 μm. (B) Schematic of discrete states of tumorigenesis in HBOW mice. Fields of recombined cells (*F*) grow at a rate (*r*) before progressing to occult dysplasia (*O*) at rate (*p*_0_). Dysplastic lesions progress to small, unpalpable tumors (*S*) at rate *p*_1_. Small lesions then progress to large, palpable tumors (*L*) at rate *p*_2_. Ordinary differential equations defining progression rates from one compartment to the next are displayed below each compartment. *K*, field size. (C) Fold difference between p95 HER2 and d16 HER2 tumors in field size (*K*), *p*_0_ rate, *p*_1_ rate and *p*_2_ rate. (D-F) Bootstrap estimates of *p*_0_ (D), *p*_1_ (E) and *p*_2_ (F). (G) Estimates of the number of occult dysplastic lesions for d16 HER2 and p95 HER2. Error bars represent 80% confidence intervals. (H) Representative whole-mouse images from two mice (left and right) with representative palpable and unpalpable tumors in boxes. Tumors were removed for digestion and transplantation into donor fatpads. The dotted lines approximate the midline. Scale bars: 10 mm. (I) Engraftment rate for tumor cells dissociated from unpalpable and palpable tumors and transplanted into donor mammary fat pads. Green denotes a successful engraftment and black denotes a transplant that failed to grow (*n*=5). The lower-right image shows a representative outgrowth from a palpable p95 tumor transplant (magenta). Scale bar: 1 mm. (J,K) Number of non-synonymous, exonic mutations from whole-exome sequencing for d16 (*n*=6) and p95 (*n*=5) tumors of any size (J) or separated by whether they were unpalpable and palpable (K). (L) Number of non-synonymous, exonic mutations comparing unpalpable tumors of any genotype (*n*=6) to palpable tumors of any genotype (*n*=5). Error bars are represented as mean±s.e.m.

Our data suggest that an ill-defined state prior to screen detection is a critical transition that a transformed cell encounters. For instance, two putative neoplastic states in the mouse mammary gland have been established: hyperplastic alveolar nodules and intraductal lesions (Cardiff et al., 1977; Cardiff and Wellings, 1999; Medina, 2000, 1979), both of which are below our sensitivity of detection using whole-mouse imaging. Confocal imaging of Crainbow fluorescence in formalin-fixed paraffin-embedded (FFPE) sections from HBOW mice identified extensive side-budding, reminiscent of hyperplastic alveolar nodules, that predominantly expressed d16 HER2, as well as lesions within the duct expressing both d16 and p95 HER2 (Fig. 4A). Owing to the rarity and labile nature of these lesions, it was exceedingly difficult to reliably and consistently quantify the number of neoplastic lesions, and, as such, we define this state as the ‘occult’. Using our mathematical model, a new compartment for occult dysplastic lesions (*O*) was incorporated to develop a four-state model of tumorigenesis (Fig. 4B).

The four-state model revealed that the transition rate out of the field compartment (*p*_0_) was roughly an order of magnitude higher for p95 than that for d16, and the transition rate into the palpable compartment (*p*_2_) was roughly twice as high for p95 compared to that for d16 (Fig. 4C,D,F, Table 2). Not present in the three-compartment (FSL) model, the transition rate from an occult dysplasia to a screen-detected neoplasia (*p*_1_) was about 2-fold greater for d16 than that for p95, although this was not significantly different (Fig. 4D,E, Table 2). We used this model to estimate the number of occult (*O*) lesions over time. Estimates of the number of occult lesions were not predictive but suggested that p95-expressing cells progress more frequently from the field (*F*) and generate more occult lesions that readily advance to screen-detectable lesions (Fig. 4G). This occurs despite a relatively small reservoir of field cells. Despite the uncertainty in the rate *p*_1_ and the state *O*, inclusion of an occult compartment highlights that the transition from a field cell to a detectable tumor is not a linear process and that each genotype encounters a critical rate-limiting step in the tumorigenic process that occurs long before the tumor becomes detectable.

**Table 2.** Four-compartment model parameters with 95% confidence intervals

Tumor size cutoff	Parameter	d16	95% c.i.	p95	95% c.i.
	*F*_0_	26,000	N/A	12,000	N/A
	*K*	233,000	N/A	26,000	N/A
	*r*	0.7313	N/A	0.1346	N/A
9 mm^2^	*p*_0_*	3.40×10^−6^	2.870×10^−6^, 4.339×10^−6^	3.20×10^−5^	2.652×10^−5^, 4.952×10^−5^
	*p*_1_	10.4332	0.4220, 65.5811	5.6362	0.2117, 15.5382
	*p*_2_*	0.0237	0.0178, 0.0310	0.0426	0.0348, 0.0522
1.5 mm^2^	*p*_0_*	3.38×10^−6^	2.861×10^−6^, 6.940×10^−6^	3.21×10^−5^	2.632×10^−5^, 5.079×10^−5^
	*p*_1_	8.2566	0.1101, 99.8215	4.7475	0.2064, 17.1339
	*p*_2_*	0.0764	0.0614, 0.0981	0.1258	0.1051, 0.1586

*F*_0_, field size at initiation (number of cells); *K*, field carrying capacity (number of cells); *r*, field replacement rate; *p*_0_, transition rate from field to occult; *p*_1_, transition rate from occult to small; *p*_2_, transition rate from small to large.

*Denotes that the 95% confidence intervals generated by bootstrapping do not overlap.

Next, we determined whether progression to malignancy was associated with transition through the occult compartment. One measure of progression is a test of autonomous growth that can be modeled by transplantation into a syngeneic host (Borowsky, 2011; Horava and Skoryna, 1955). Whole-mouse imaging was performed to identify paired palpable and unpalpable tumors of the same genotype from the same mouse (*n*=5 per genotype). These tumors were then dissected and transplanted into the orthotopic fatpad of immune intact and syngeneic FVB/N female mice between 4 and 8 weeks of age (Fig. 4H). Successful engraftment was determined by a tumor growing to humane endpoint and maintaining HER2 expression as confirmed by whole-mouse imaging. Evaluation of the engraftment rate revealed that all palpable p95 tumors successfully engrafted, whereas 80% of d16 tumors transplanted successfully (Fig. 4I), suggesting again that, at clinical diagnosis, both d16 and p95 tumors had progressed to an autonomous state. 40% of p95 unpalpable lesions transplanted successfully, whereas none of the d16 unpalpable lesions could be transplanted (Fig. 4I). This suggests that progression to malignancy occurs very rarely for unpalpable d16 HER2 tumors, whereas p95 tumors can progress while transitioning from the occult stage, resulting in nascent lethal lesions at screen detection.

The multistep model of tumorigenesis suggests that progression is linked to the accumulation of somatic mutations, indicating that the differences in transition rate between isoforms could be due to the rate of somatic mutation in HBOW tumors (Balmain and Harris, 2000; Fearon and Vogelstein, 1990). To test this, whole-exome sequencing was performed on palpable and unpalpable tumors for both p95 and d16 to assess mutational burden. On average, d16 tumors of any size had 5111 non-synonymous, exonic mutations, whereas p95 tumors had 3784 mutations (Fig. 4J, Table 3). Once separated by size, there was no significant difference between d16 palpable tumors (*n*=3) with 2792 mutations and p95 palpable tumors (*n*=2) with 2937 mutations on average (Fig. 4K). There was an average of 7430 mutations in d16 unpalpable tumors (*n*=3) and 4348 mutations in p95 unpalpable tumors (*n*=3) (Fig. 4K). Comparison of unpalpable and palpable tumors regardless of genotype further reinforces a trend towards less mutational burden in palpable tumors (5889 mutations) compared to unpalpable tumors (2850 mutations) (Fig. 4L). This suggests that somatic mutation rate is independent of HER2 isoform expression and occurs prior to screen detection.

**Table 3.** Number of each mutation type per tumor from whole-exome sequencing

Genotype	Size	Number of mutations by type							
		Missense	Nonsense	Start codon	Start loss	Stop loss	Frameshift	In frame	Total
**Mouse 1**									
d16	Palpable	3830	610	0	10	4	5	0	4459
d16	Palpable	2631	254	0	4	0	5	1	2895
d16	Unpalpable	3443	259	0	3	1	3	1	3710
p95	Unpalpable	4580	390	0	12	1	3	0	4986
p95	Unpalpable	4493	440	0	11	0	6	1	4951
**Mouse 2**									
d16	Palpable	950	69	0	2	1	1	0	1023
d16	Unpalpable	6184	617	0	13	2	1	0	6817
d16	Unpalpable	10,878	857	2	20	3	5	0	11,765
p95	Palpable	4090	306	0	9	3	1	0	4409
p95	Palpable	1347	115	1	1	0	1	0	1465
p95	Unpalpable	2856	244	0	6	2	1	0	3109

**Fig. 5.**
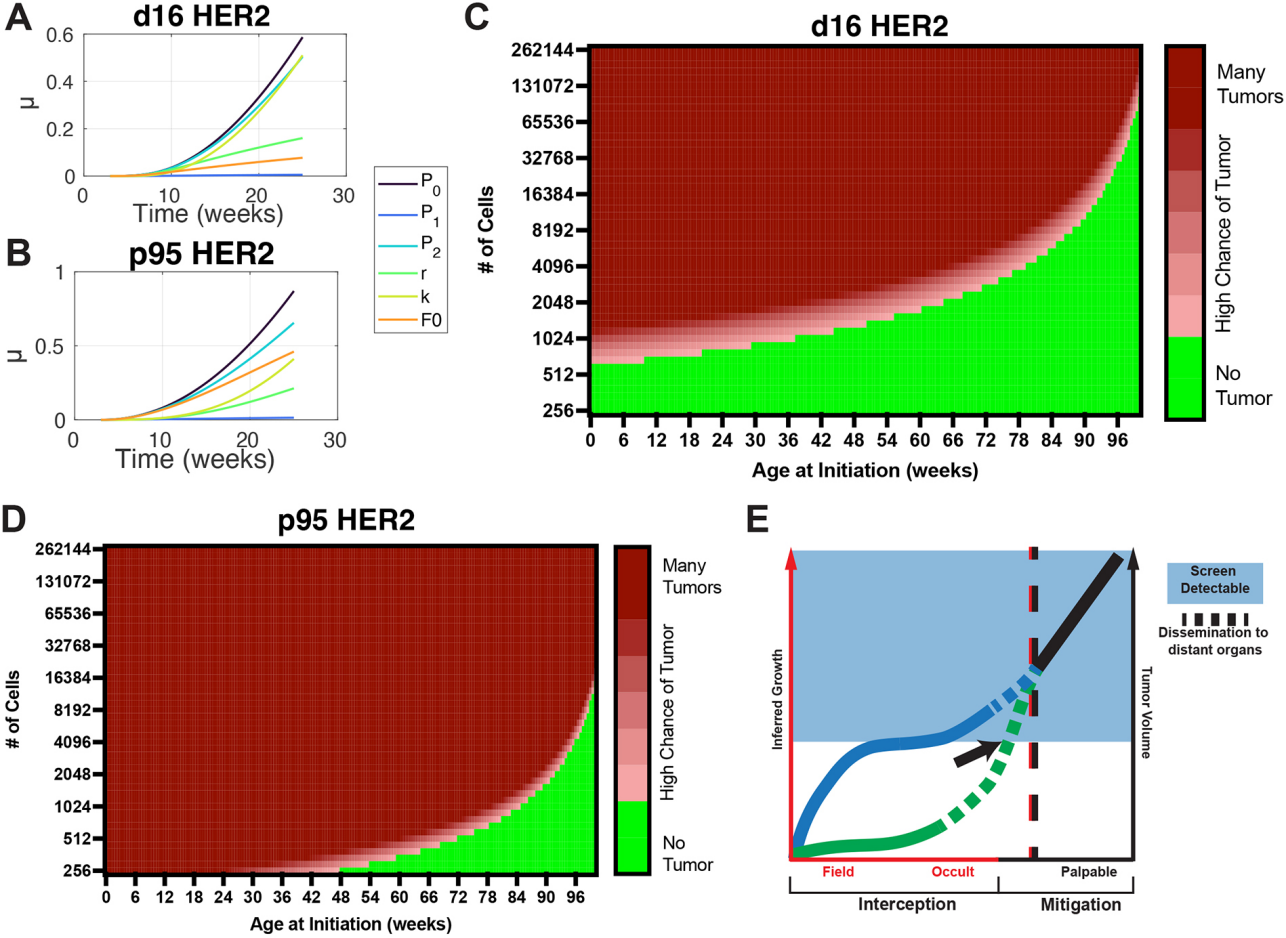
**Clinical implications of Crainbow modeling.** (A,B) Sensitivity (μ) plots of the model fit showing the relative contribution of each variable to the uncertainty in determining the transition to palpable tumors expressing d16 (A) and p95 (B). (C,D) Simulated data using the three-compartment (FSL) model to visualize the impact of changing both the time of initiation and field size on the likelihood of a tumor forming within the lifetime of a mouse. The simulated data are based on oncogenic cells expressing either d16 (C) or p95 (D). Green represents a low likelihood of any tumor forming (<0.5). The red gradient represents an increasing likelihood of a tumor developing (0.5-1). Dark red represents one or more tumors. (E) Graphical representation of two cases of non-linear tumor progression. Both cases occur prior to the continuous, clinically observable growth of large tumors. The blue line represents a rapidly growing field and its progression to a small screen-detectable tumor. In this case, cancer mitigation by screen detection (blue shading) can occur before dissemination to distant organs (dotted line) and reduce the risk of metastasis. In contrast, the green line represents the slow-growing field that rapidly progresses to an invasive lesion that disseminates (dotted line) before becoming screen detectable (black arrow). This nascent lethal phenotype reduces the efficacy of mitigation. Thus, cancer-interception strategies must be pursued to prevent nascent lethal progression.

Lastly, sensitivity analysis was performed in order to understand how variations in parameters (i.e. *r*, *K*, *p*_1_, *p*_2_) affect overall tumorigenic potential (Dickinson and Gelinas, 1976; Morris, 1991). In this analysis, we observed that the most sensitive parameter for a clinically detectable tumor (*L*) is *p*_0_ – the transition rate from the field to an occult dysplastic lesion – for both the p95 and d16 models (Fig. 5A,B). In other words, changes in the occult transition rate have major impacts on tumorigenic potential. An interesting difference between the genotypes is that for d16, the field carrying capacity (*K*) has effects of the same magnitude of the occult transition (*p*_0_) (Fig. S1A,B), whereas for p95, *K* and the initial field size (*F*_0_) are about equal and have half the magnitude compared to *p*_0_. This suggests that the development of p95 tumors is less dependent upon the carrying capacity (*K*) and the initial field size (*F*_0_) compared to the development of d16 tumors.

We can visualize differences in occult transitions by leveraging the statistical models for d16 or p95 tumor development to simulate the number of palpable tumors that would form from a given field of cells within the average lifetime of a captive mouse (2 years). If the initial event occurs early (∼3 weeks of age), many detectable p95 tumors are likely to develop from a very small field of cells (<256) compared to d16 HER2 tumors, for which nearly 1000 cells are required to have a high probability of one tumor (Fig. 5C,D). If the simulation begins at 1 year of age, in order to have a high likelihood of one tumor, d16 HER2 requires a field of oncogenic cells that is nearly six times larger than that required by p95 HER2. This difference in the number of cells needed to likely form a tumor is representative of the relative frequency at which any oncogenic cell is able to navigate through the relatively difficult occult transition to eventually become a large, invasive tumor. This projection of our data demonstrates how nonlinear progression to an invasive tumor during the occult transition can lead to nascent lethal cancers that confound current cancer-screening paradigms.

## DISCUSSION

The benefits and risks of cancer screening are under increasing scrutiny, and, in the breast, there is evidence to suggest net neutrality in survival despite decades of intense screening (Welch and Dey, 2023). Three main phases of cancer intervention have recently been summarized and include prevention, interception and mitigation (Castle et al., 2024; Domchek and Vonderheide, 2024). The conventional linear progression paradigm supports the strategy of mitigation, using surveillance to detect anatomically small tumors, with the expectation that clinically detecting and treating these smaller tumors results in decreased mortality (Armitage and Doll, 1954; Fearon and Vogelstein, 1990; Nordling, 1953). Unfortunately, even after decades of screening for clinically detectable tumors, this strategy has not resulted in the expected positive benefits of reducing mortality or late-stage disease (Welch et al., 2019).

Our data demonstrate the paradox of cancer screening using a genetically tractable mouse model system (Fig. 5E). We show that tumors can originate from rapidly growing premalignant fields (i.e. d16 HER2). Larger field sizes increase the likelihood that a rare clone advances to a small image-detectable event that is unlikely to disseminate in the mouse but, if left untreated, may eventually metastasize. Therefore, early screening and appropriate interventions could reduce the likelihood of a malignant transition and prevent recurrent, late-stage disease. In contrast, our modeling also shows how quiescent clones (i.e. p95 HER2) are less likely to rapidly expand. Yet, their ability to transition from premalignancy to malignancy is high. Thus, these nascent lethal clones would already have disseminated and are much more likely to escape prior to anatomic disruption sufficient for clinical detection. In this case, interception methods, which, if able to predict when and where cancer progression will occur, can treat the tumor before the transition to a screen-detectable lesion, may be more successful than traditional mitigation (Castle et al., 2024; Domchek and Vonderheide, 2024).

We also provide experimental evidence for a model of ‘biological predeterminism’ (Macdonald, 1966, 1951), wherein the lengthy preclinical process establishes lethality prior to detection. Curiously, the most detectable lesions in our model were associated with an isoform of HER2 that is also predictive of favorable therapeutic responses (Castagnoli et al., 2014). Thus, d16-driven tumors are likely to have favorable outcomes regardless of early detection. In contrast, a tumor lacking a treatable domain, such as p95 HER2 tumors (Abraham et al., 2019; Pedersen et al., 2009; Scaltriti et al., 2007), is also the most unlikely to be detected and most likely to disseminate. Thus, in our model, p95 tumors represent the worst-case scenario for a patient – a treatment-insensitive tumor that is not screen detectable. This continues to support the notion that p95 tumors are associated with worse clinical outcomes (Abraham et al., 2019; Carvajal-Hausdorf et al., 2015; Scaltriti et al., 2007) and further warrant the need to clinically validate the p95 isoform as a predictive biomarker.

Our model points to an important occult transition in which a transformed cell establishes a neoplastic lesion. We chose the term occult because of the inability to image the exact moment of this transition. This leads to a challenging question: what is the occult transition? The occult transition could represent a location in the form of a privileged site of growth. We speculate that an occult compartment could reside inside the duct, similar to the ductal hyperplasias described several decades ago (Medina, 2000), in which growth inside the duct provides for a unique microenvironment compared to growth into the surrounding fat. For instance, mammary ducts are surrounded by a layer of ductal macrophages (Dawson et al., 2020). This could provide a mechanism wherein disseminating cells could be in close proximity to macrophage populations necessary for early invasion (Linde et al., 2018; Sidani et al., 2006). The occult transition could also represent a moment in time when malignancy is established by changes in the host that impact the fitness of tumor cell phenotypes. These could be systemic in nature and may range from immune ageing, microbiome changes, obesity, injury and many others (Swanton et al., 2024). The occult transition could also occur through oncogene-driven differences in cell state plasticity. We also have evidence for early-state changes in the p95-expressing mammary epithelium that result in epithelial–mesenchymal transition-like states and an early invasive histological phenotype (Ginzel et al., 2021). This suggests that functionally selective signaling across HER2 isoforms is also important (Dankort et al., 2001). The benefit of our model lies in the full accounting of the natural history of these isoform-driven tumors in an untreated, immune intact mouse, which provides a future benchmark for analyzing how the various cell-intrinsic and -extrinsic processes contribute to not only the occult transition, but also each aspect of tumor development.

There are limitations and assumptions to our models that must also be considered. A significant assumption is the cutoff between a screen-detectable and palpable tumor in imaging-based data. Although somewhat experimentally determined from palpation data, we tested this cutoff by using a previously described, anatomically based 1.5 mm^2^ cutoff (Ginzel et al., 2021). When applied to our four-compartment model, parameter estimates changed by less than 10% and the overall tumor transition dynamics were unchanged due to the high impact of the *p*_0_ transition in relation to the *p*_2_ transition (Table 2, Fig. S2). Although our experimental HBOW model seeks to recapitulate the isoform heterogeneity in patients, our system is an abstraction of a much more complicated tumor ecosystem. One limitation to our approach is that we were unable to determine interactions between isoforms and extrinsic immune responses due to the lack of single-genotype controls. Secondly, our mouse model overexpresses human HER2 isoforms, and although this is a useful model system of *HER2* amplification, it is still not entirely naturalistic. Similarly, the system of ODEs is a mathematical abstraction of the complex tumor ecosystem and has several key limitations, some of which would be common to any similar mathematical model. Primarily, spatial relationships cannot be described by the current ODEs. Additionally, the parameters of the dynamical system are estimated using survey data (mouse necropsy) instead of longitudinal data. Thus, the use of certain statistical frameworks, e.g. nonlinear mixed-effects modeling, that describe the variation between individuals are not applicable (Comets et al., 2017; Pinheiro and Bates, 1995). However, further analysis that is outside the scope of this investigation would provide error bars for the parameters *r*, *K* and *F*_0_ while quantifying the uncertainty in all parameters. Also, the equation for *L* (large, palpable tumors) is limited to the short time from when they are first detectable to the humane lifespan of the mouse and, therefore, does not include any long-term dynamics. This is in comparison to clinical tumors that would include treatment and much lengthier timescales. Lastly, the models describe observations of two-dimensional tumor size (in mm^2^), not the biological state (e.g. whether tumors are metastatic or benign) or interactions between tumor genotypes. Addressing these limitations to more deeply understand these transitions critical to tumor development are paramount to improving the ability to intercept nascent lethal lesions.

Intercepting the progression of a premalignant clone is a significant challenge but could provide a transformative option for preventing cancer. Overall, we demonstrate the importance of reevaluating major conceptual advances of the past with modern-era technological advances. Our work affirms the rules of progression yet provides an experimental framework for future work for predicting progression risk in a patient's lifetime.

## MATERIALS AND METHODS

### Animal studies

All animal studies were performed according to an approved protocol from the Duke Institutional Animal Care and Use Committee. HER2 Crainbow mice were crossed to MMTV Cre (MMTV-Cre/Line D; Mouse Genome Informatics, #3581599, RRID:MGI:3581599) to generate HBOW mice (Ginzel et al., 2021). Both lines were fully backcrossed to the FVB/N background. All experimental animals were pre-emptively enucleated at 3 weeks of age due to off-target harderian gland tumors (Garner et al., 2022). All experimental animals used were female and between the age of 3 and 28 weeks.

### Mammary gland epithelial cell analysis

Digestion of mammary glands for analysis of epithelial cell number was performed following the protocol from Ludwik et al. (2021). Briefly, all ten mammary fat pads were removed from HBOW mice, finely minced, and incubated in collagenase A (2 mg/ml, Roche, 10103578001) in RPMI medium (Gibco, 11875093) for 3 h at 37°C with 5% CO_2_. Next, the digested tissue was incubated with DNase I, followed by incubation in red blood cell lysis buffer (Sigma-Aldrich, R7757) on ice for 2 min. Cells were then washed with 10 ml of 1× PBS. The digested tissue was then spun down and resuspended in Accumax (Invitrogen, 00–4666–56) for 10 min on a rotating shaker at 37°C. Next, cells were pelleted and resuspended in 5× trypsin (Thermo Fisher Scientific, 15400054) for 5 min at 37°C, before being filtered through a 40 µm filter. Samples of single-cell suspensions were imaged on a Zeiss LSM880 confocal microscope and counted using Imaris software (v10.1).

### Whole-mouse imaging

Mice were euthanized with CO_2_ and pinned on vinyl dissecting pads to expose all ten mammary glands. Each mouse was then imaged on a Zeiss Axio Zoom microscope. Images were imported into either QuPath (Bankhead et al., 2017) or ImageJ2 (Rueden et al., 2017) for manual measurement and classification of all tumors.

### Whole-organ imaging

Mammary glands and lungs were dissected and placed in or inflated with 10% neutral buffered formalin (NBF) for fixation. After 2 h of fixation, tissues were briefly washed with 0.5% Tween 20 in PBS for 30 min, before being submerged in FUnGI clearing solution (Rios et al., 2019) for at least 4 h. After clearing, tissues were sandwiched between a glass slide and coverslip for imaging on a Zeiss LSM880 confocal microscope. For whole-lung imaging, spectra were obtained from lambda-mode imaging for each Crainbow fluorescent protein and stored as ‘online fingerprints’ for imaging. This process ensured that only Crainbow fluorescence was captured and reduced background fluorescence. Whole lungs were removed from FUnGI clearing solution and rehydrated in PBS before paraffin embedding and sectioning for H&E staining and whole-slide imaging. Imaging of occult lesions was done on FFPE sections of mammary glands fixed in NBF. FFPE sections were deparaffinized and mounted with DAPI nuclear stain, before imaging on a Zeiss LSM880 confocal microscope. Crainbow fluorescence is imageable following FFPE processing with no further amplification or staining (Ginzel et al., 2021). Analysis was performed using ImageJ2 (Rueden et al., 2017) and Imaris software (v10.1).

### Mathematical modeling

#### Sensitivity analysis

Sensitivity analysis is an analytical technique that explains the uncertainty in the model output as a function of the uncertainty in the model parameters. Sensitivity analysis provides insight into the relationship between the parameters (*r*, *K*, *p*_1_, *p*_2_) and the values of the states (*F*, *S* and *L*). The more sensitive a parameter is, the more effect it has on the state value. If a parameter is ‘insensitive’, large changes in its value result in small changes to the state values. Here, the Morris method (Morris, 1991) was employed to screen the parameters of the system, using the SAFE software package in MATLAB (v9.14; 2023a) (Pianosi et al., 2015). Morris screening varies one parameter at a time while the others remain constant, and an ‘elementary effect’ is calculated. The elementary effect is the absolute difference in the change in state, divided by the change in the parameter value. The elementary effect is computed at each point in time and the result is plotted.

#### Parameter estimation

The parameters for the logistic equation describing the field (*r*, *K* and the initial condition *F*_0_) were estimated first using mammary gland digestion data (Figs 2B and 3C). Ordinary least squares (OLS) regression was used to fit the solution of the logistic equation to the week 3, 4 and 6 data. The MATLAB function ‘lsqcurvefit’ was used to find the values *r*, *K* and *F*_0_ that minimize the least-squared error between the data points and the solution of the logistic equation. For each of the estimated parameters, we set a range of allowable values and the ranges were chosen to be roughly the same as the observed data. The time coordinates were shifted so that the initial condition *F*_0_ corresponded to an age of 3 weeks, the time of the earliest digestion studies. We assumed that growth of the mammary duct was essentially complete by 6 weeks of age, so we chose the upper and lower bounds of *K* according to the range of data at 6 weeks. After deriving estimates of the logistic growth parameters of the field, these values were set and parameter estimates of the transition rates in the *S* and *L* compartments, *p*_1_ and *p*_2_, respectively, were calculated [and *p*_0_ in the four-compartment (FOSL) model]. The state transition rates were also estimated by OLS regression using the MATLAB function lsqcurvefit. Here, we solved the ODEs (Figs 3B and 4C) using the ‘ode15s’ function in MATLAB and then minimized the sum-of-squared differences with the mouse survey data. Every mouse produced four data points, namely, *S* and *L* for both p95 and d16 tumors, and the parameters for the p95 and d16 models were fitted independently. To produce confidence intervals for the estimations, we employed a hierarchical bootstrap method. There were 120 mice in the survey dataset and we sampled *n*=120 mice with replacement. Then, from each sampled mouse, we resampled the tumors in that mouse, recording the area of each resampled tumor. The resampled tumors of this synthetic mouse were then stratified by genotype and state (*S* or *L*) so that each sampled mouse produced four data points. The hierarchical bootstrap was repeated 1000 times. Parameters were estimated using median values and confidence intervals are given by percentiles of the bootstrapped distribution.

To account for additional uncertainty, the field parameters *r*, *K* and *F*_0_ were resampled at each bootstrap iteration, using a normal distribution with the mean given by the preliminary OLS estimate and standard deviation equal to 5% of the mean. Uncertainty in the mouse age was modeled by adding a normally distributed random number with a mean of 0 and standard deviation of 0.05 (weeks) to the age of each synthetic mouse at every bootstrap iteration.

#### Simulations

To produce the results in Fig. 5C,D, we used the parameter estimates for *p*_1_ and *p*_2_ in the FSL model and forward simulated the system from the age at initiation, given on the *x*-axis, to 2 years, the expected lifespan of a mouse. Here, *K* was set to the observed number of transformed cells (given on the *y*-axis) so that the growth of the field (*F*) was not considered as part of the simulation results.

### Transplant studies

Tumors to be used for transplant were selected from Axio Zoom imaging and digested following the same digestion protocol as described in the ‘Mammary gland epithelial cell analysis’ section above. Single cells were resuspended in USP saline (Vedco, NDC 50989-885-17). Female FVBN/J mice (The Jackson Laboratory) between 4 and 8 weeks of age were anesthetized using isoflurane and ∼500,000 isolated tumor cells were injected into the right, fourth (inguinal) mammary gland. Tumors were measured once weekly. At tumor burden endpoint, whole-mouse imaging was performed as described above to assess genotype.

### Whole-exome sequencing

All tumors identified by whole-mouse imaging along with liver tissue were dissected and flash frozen. DNA was isolated and exome libraries were prepared with the Twist Exome Kit (Twist Bioscience). Sequencing and advanced bioinformatic data analysis were performed by MedGenome (Foster City, CA, USA) following a standardized analysis pipeline.

## Supplementary Material

10.1242/dmm.052113_sup1 Supplementary information
