# Supplementary material for: Nonlinear progression during the occult transition establishes cancer lethality
Source: Dis Model Mech. 2025 Mar 19;18(3):dmm052113. doi: 10.1242/dmm.052113 (PMC11957451; doi:10.1242/dmm.052113)
Supplement: Supplementary information [file dmm-18-052113-s1.pdf]

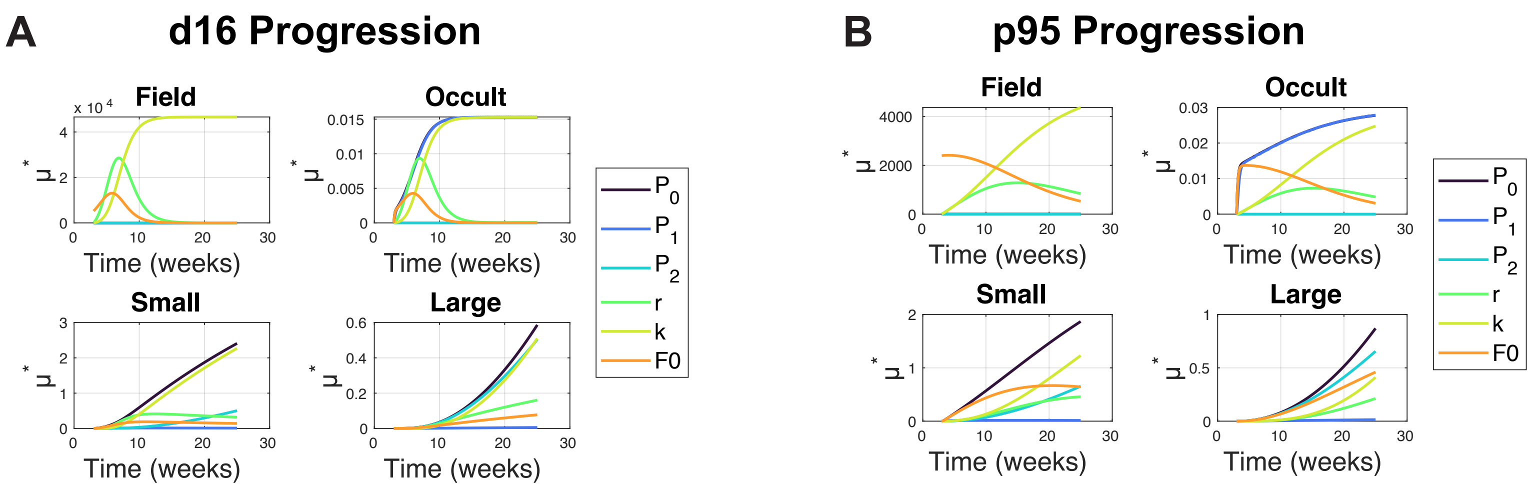

Fig. S1. Sensitivity analysis of the four compartment model. A-B, Sensitivity plots for each parameter of the four compartment model for d16(A) and p95(B).

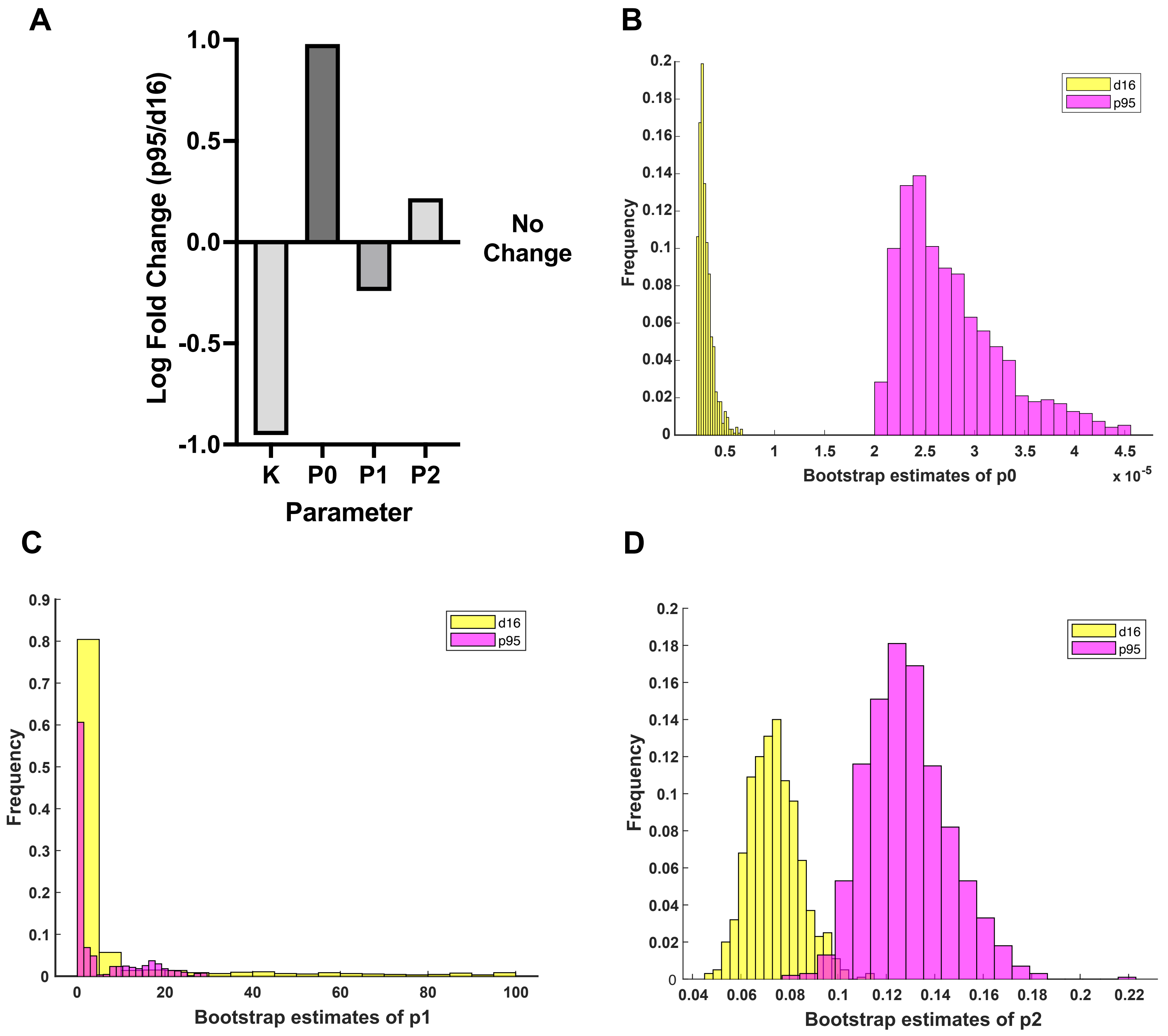

Fig. S2. Effect of S:L cutoff in four compartment model of tumorigenesis. A, Fold change difference in parameter estimates between p95 and d16 when the cutoff from S:L is 1.5mm2. **B-D,** Bootstrap estimates of p0(B), p1(C), and p2(D).
